# Western Blot Analysis of *Leishmania infantum* Antigens in Sera of Patients with Visceral Leishmaniasis

**Published:** 2019

**Authors:** Soudabeh HEIDARI, Javad GHARECHAHI, Mehdi MOHEBALI, Behnaz AKHOUNDI, Shahab MIRSHAHVALADI, Bahareh AZARIAN, Homa HAJJARAN

**Affiliations:** 1. Department of Medical Parasitology and Mycology, School of Public Health, Tehran University of Medical Sciences, Tehran, Iran; 2. Human Genetics Research Center, Baqiyatallah University of Medical Sciences, Tehran, Iran; 3. Department of Molecular Systems Biology at Cell Science Research Center, Royan Institute for Stem Cell Biology and Technology, ACECR, Tehran, Iran; 4. Protein Chemistry Unit, Biotechnology Research Center, Pasteur Institute of Iran, Tehran, Iran

**Keywords:** Visceral leishmaniasis, Human, *Leishmania infantum*, Antigen, Western blotting

## Abstract

**Background:**

Visceral leishmaniasis (VL) is endemic in the northwest and south of Iran. Untreated cases of VL could cause death. The aim of the present study was to evaluate the diagnostic performance of western blotting to detect a specific immunodominant proteins pattern for *Leishmania infantum* infection using human sera infected with VL.

**Methods:**

We studied a panel of 122 cryopreserved human serum samples from the leishmaniasis Research Laboratory, Tehran University of Medical Sciences, Tehran, Iran from 2010 to 2017.

Serum samples were collected from visceral (Group I, n: 43) and cutaneous leishmaniasis (CL) (Group II, n: 8) patients, healthy individuals from endemic (Group III, n: 13) and non-endemic (Group IV, n: 16) areas for VL, and patients with other infectious diseases (Group V, n: 42). Total antigens were prepared from the Iranian strain of *L. infantum* promastigote form.

**Results:**

In western blotting method, 34 protein bands of 14 to 163 kDa were recognized using the sera of VL patients. The polypeptide fractions with the highest frequency including 29, 51, and 62 kDa fractions were detected using 81.4%, 79%, and 81.4% of the sera, respectively. These bands were not detected using the sera of the negative control. Moreover, 19–23, 27, 31–35, 143–163, and 109 kDa fractions were detected specifically using the sera of the patients with VL.

**Conclusion:**

This technique could be a primary step for further exploration of VL immunodominant antigens for cloning (or any technique) further investigations for future planning.

## Introduction

Leishmaniasis is a parasitic disease found in some parts of tropical and subtropical countries in the world, including Iran. Leishmaniasis is caused by the parasitic protozoa transmitted by the bite of infected female phlebotomine sandflies. There are 3 main forms of the diseases – visceral (also known as kala-azar), cutaneous, and muco-cutaneous leishmaniasis (1, 2). In Iran, both forms of cutaneous and visceral leishmaniasis can be found. Cutaneous leishmaniasis (CL) is caused by *Leishmania tropica* and *L. major* (3). The Mediterranean form of visceral leishmaniasis (VL) is caused by *L. infantum.* VL is endemic in the northwest and south of Iran and more than 3000 cases were recorded as serology and parasitology confirmed cases from 1996 to 2010 in Iran (4). The disease is characterized by irregular fever, weight loss, hepatosplenomegaly, and anemia is fatal if left untreated in over 95% of cases (5).

In VL, diagnosis is made by combining clinical signs with parasitological or serological tests (6). The diagnosis is confirmed by observing amastigotes in bone marrow, liver, spleen or lymph node biopsies, although it is an aggressive method. Parasitological methods with high specificity and variable sensitivity may be used as the method of choice for the diagnosis of VL. These methods are may be suitable during the acute phase of VL, but are not effective in the chronic and asymptomatic cases characterized by lower parasitemia and presence of specific antibodies in the serum or plasma (7, 8).

Several serological methods have been proposed like the direct agglutination test (DAT), indirect immunofluorescence assay (IFA), and ELISA. None of the methods for the diagnosis of VL can be regarded as 100% safe, especially in asymptomatic and chronic VL case (9–11).

However, several studies have presented valuable information about the parasite anti-genic profile using alternative diagnostic sero-logical methods like western blot (WB) (12, 13). Two primary advantages of WB are sensitivity and specificity. Western blotting (also called immunoblotting) can detect as little as 0.1 ng/mm^2^ of protein from a parasite, virus, or bacteria in a patient (14).

In the present study, we used immunoblotting technique with total protein antigen extracted of *L. infantum* for detection of specific antibodies in patients infected with VL in the acute and chronic phase of the disease as well as cases infected with CL and other infectious diseases. The western blot method may be used as an alternative method for detecting specific antibodies against *L. infantum* antigenic polypeptides to confirm the laboratory diagnosis of VL.

## Materials and Methods

### Sampling

We studied a panel of 122 cryopreserved human serum samples from the leishmaniasis Research Laboratory, Tehran University of Medical Sciences from 2010 to 2017.

Serum samples were divided into five groups:

Group I: Sera that were clinical, parasitologically, and serologically (DAT) positive (n: 43) for VL from endemic (Ardebil, East Azerbaijani) and non-endemic (Kerman, Gilan and Semnan) provinces for VL.
Group II: Sera that were clinically and parasitologically positive (n: 8) for CL.
Group III: Sera of blood donors (n: 13) without any clinical signs or symptoms that were DAT negative for VL. The donors were from an endemic area (Ardebil, Iran) and the samples were used as negative controls.
Group IV: Sera of blood donors (n: 16) without any clinical signs or symptoms that were DAT negative for VL. The donors were from a non-endemic area (Tehran, Iran) and the samples were used as negative controls.
Group V: Sera collected from patients with other infectious or autoimmune disease (n: 42) that were DAT negative for VL; malaria (n:5), toxoplasmosis (n:8), fascioliasis (n:3), strongyloidiasis (n:3), hydatidosis (n:2), taeniasis (n:1), trichostrongylosis (n:1), toxocariasis (n:1), antinuclear antibodies (n:2), rheumatoid factor (n:4), syphilis (n:1), brucellosis (n:3), rubella (n:5) and cytomegalovirus (n:3).

For positive and negative control, we used one pooled sera including 5 serologically and parasitological positive cases for VL and 5 serologically and parasitological negative cases for VL from endemic and non-endemic areas for VL, respectively. This study was approved by the Ethics Committee of the Tehran University of Medical Sciences, Tehran, Iran No: IR-TUMSVCR-REC-1395-721.

### Antigen preparation

The antigen used was the total extract of *L. infantum* promastigotes (Iranian strain: MCAN/IR/14/M14) were grown at 24 °C in the RPMI 1640 medium (Gibco, USA) supplemented with 10% heat-inactivated fetal calf serum and were harvested at the late log phase and early stationary phase of growth. The cells were washed with phosphate buffered saline three times (pH 7.4). The parasitic concentration was adjusted to 3×10^8^ promastigotes/mL.

Proteins were extracted using trichloroacetic acid (TCA) in acetone (15, 16). Briefly, the cells were suspended in 10% TCA in acetone supplemented with 0.07% (w/v) dithiothreitol (DTT). Then, they were incubated at −20 °C for 1 h. The samples were then centrifuged at 26000 g for 20 min at 4 °C and the pellets were washed with cold acetone containing 0.07% (w/v) DTT, incubated at −20 °C for 1 h and centrifuged at 4 °C. Washing and sedimentation of the pellets repeated three times. Residual acetone was removed by air drying. The pellets were resolubilized in sample lysis buffer containing 8.4 M urea, 2.4 M thiourea, 50 mM DTT, 2% ampholyte pH 3–10, 5% CHAPS detergent, 1% Triton x-100, 1% DNase, 0.25% RNase, 1 mM PMSF, protease inhibitor cocktail (Roche, Germany). The protein concentration was determined by Bradford assay using bovine serum albumin as the standard (17). The extracted proteins were stored at −70 °C until use.

### Sodium dodecyl sulfate-polyacrylamide gel electrophoresis

The samples were electrophoresed on 1-mm thick slab gels with a 12% polyacrylamide in the running gel, and a stacking gel of 5% acrylamide, using a discontinuous SDS buffer system. The antigens were diluted in sample buffer (125 mM Tris-HCl [pH 6.8], 4% sodium dodecyl sulphate [SDS], 25% glycerol, 5% (vol/vol) 2-mercaptoethanol, 0.02% bromophenol blue).

The molecular weight markers (Thermo scientific, cat no: 26610, USA) and antigens were boiled for 5 min and applied to the gel. The antigen (15μg/well) was electrophoresed using Mini-Protean II electrophoresis cells (Bio-Rad, USA). The proteins were visualized using the coomassie brilliant blue (R 250).

### Western blotting technique

The proteins from unstained polyacrylamide gels were electro-transferred onto a 0.45 μm-pore-size polyvinylidene difluoride (PVDF) membrane (GE Healthcare, USA) at 70 V (300 mA) at 4 °C for 1.5 h. The PVDF membrane was cut into vertical strips. The strips were treated with blocking buffer [5% defatted milk, 0.05% Tween 20 in tris-buffered saline (TBS), pH 7.5], at 4 °C overnight with constant shaking, and then rinsed with TBS. The membranes were treated with serum samples of different study groups diluted 1:6000 in blocking buffer at RT for 2 h with constant shaking. After washing three times with 0.05% TBS_Tween, the strips were treated at RT, for 1 h with a peroxidase-labeled affinity purified rabbit anti-human IgG H&L conjugate (Abcam Chemical Co (ab6759)) diluted 1:25000 in blocking buffer. After additional washings, bound conjugates were detected using the ECL prime western blotting detection reagent (Amersham/ GE Healthcare, Buckinghamshire, UK) and proteins were visualized by autoradiography (Image Station 4000MM PRO, Kodak, USA).

### Statistical analysis

The presence and absence and the relative molecular weight of immune-reactive proteins in blots were determined using the Quantity-one 4.6.6 software (Bio-Rad, Hercules, CA, USA).

The significant differences in banding patterns between case and control groups were evaluated by the chi-square and Fisher’s exact tests using the SAS software (version 9.3, SAS Institute, Inc., Cary, North Carolina). A *P-*value<0.05 was considered statistically significant.

## Results

### Gel electrophoresis

**Fig. 1:**
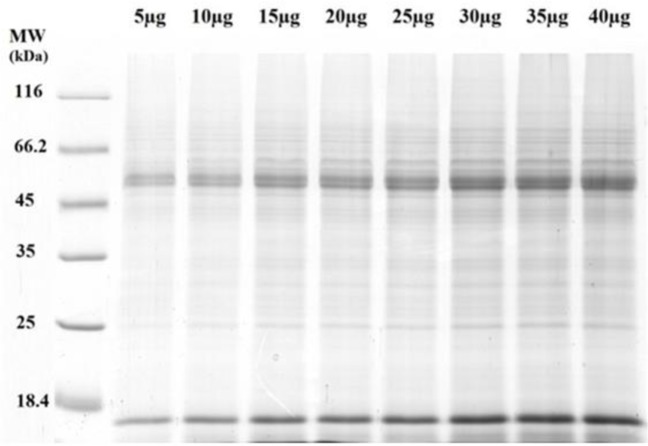
Total protein antigen of *Leishmania infantum* was separated in a 12% SDS-PAGE. MW, molecular weight of protein marker (MW: 18.4 to 116 kDa); μg, microgram

More than 40 protein bands were observed in the *L. infantum* protein profile whose molecular weight varied from 14 to <116 kDa. They were separated on SDS-PAGE 12% stained with coomassie brilliant blue R 250 (Fig. 1).

### Western blotting analysis

**Fig. 2:**
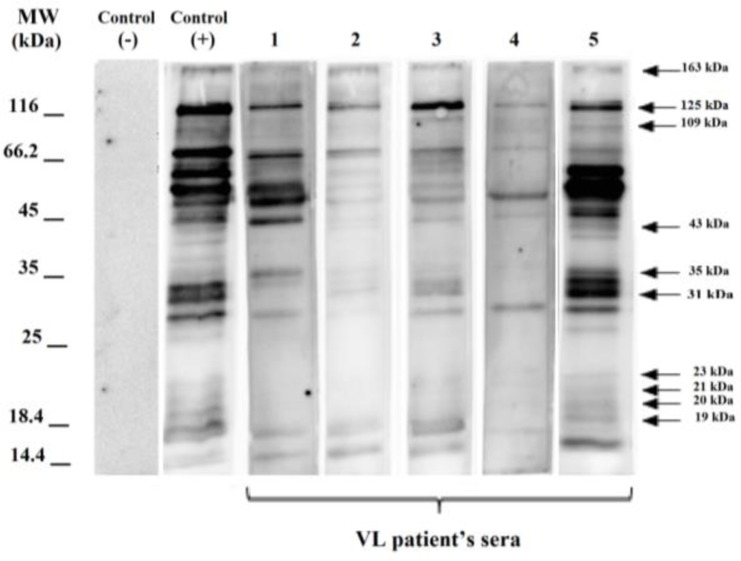
Immunoblotting of serum samples from visceral leishmaniasis (VL) patients with total antigen of *L. infantum*. (MW: 14.4 to 116 kDa); Lanes 1–5 VL patients’ sera

**Fig. 3:**
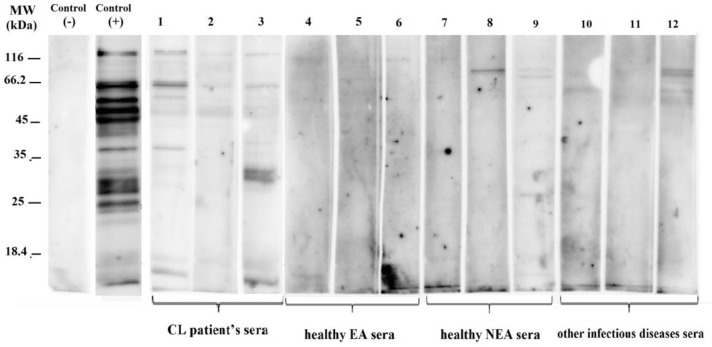
Immunoblotting of serum samples from cutaneous leishmaniasis (CL) patients, healthy individual from endemic and non-endemic areas and other infectious diseases' sera with total antigen of *L. infantum*. (MW: 18.4 to 116 kDa); Lanes 1–3 CL patients’ sera. Lanes 4–6 Healthy endemic areas (EA) sera; lane 7–9 Healthy non endemic areas (NEA) sera and lane 10–12 other infectious diseases sera

In the immunoblotting approach, the total protein antigen transferred to the PVDF membrane. The immunodominant polypeptide fractions of *L. infantum* were identified using different serum samples with diverse severities and frequencies (Figs. 2, 3).

The mean number (X ± SD) of polypeptide fractions identified using the sera of various groups was 15.37 ± 8.23 for VL (G: I), 7.75 ± 4.89 for CL (G: II), 0.69 ± 1.03 for healthy controls from endemic areas (G: III), 0.38 ± 1.02 for healthy controls from non-endemic areas (G: IV), and 1.55 ± 2.23 for other infectious or autoimmune diseases (G: V). In the western blotting method, the number of immunodominant bands was different in VL patients group, depending on the severity and titer of the antibody in serum samples.

At least one immunodominant band was identified in each sample. Overall, there was a similar pattern of reactivity between the antigen and antibody in patients with VL and 34 bands (14–163) kDa were observed with various severities and frequencies (Table 1).

**Table 1:** Frequency of *L. infantum* antigens recognized by sera from visceral leishmaniasis patients (VL, n: 43), cutaneous leishmaniasis patients (CL, n: 8), sera from healthy individual from endemic (EA, n: 13) and non-endemic (NE, n: 16) areas for visceral leishmaniasis and other infectious diseases sera (n: 42)

***Proteins (kDa) No:34 bands***	***Group I (VL) (N=43; %)***	***Group II (CL) (N=8; %)***	***Group III (healthy EA) (N=13; %)***	***Group IV (healthy NEA) (N=16; %)***	***Group V (other diseases) (N=42; %)***
**14**	27.91	50.00	0.00	0.00	2.38
**17**	62.79	37.50	7.69	6.25	0.00
**19**	20.93	0.00	0.00	0.00	0.00
**20**	23.26	0.00	0.00	0.00	0.00
**21**	27.91	0.00	0.00	0.00	0.00
**23**	4.65	0.00	0.00	0.00	0.00
**26**	32.56	0.00	0.00	6.25	2.38
**27**	13.95	0.00	0.00	0.00	0.00
**29**	81.40	25.00	0.00	0.00	4.76
**31**	65.12	25.00	0.00	0.00	0.00
**33**	72.09	25.00	0.00	0.00	0.00
**35**	53.49	0.00	0.00	0.00	0.00
**38**	37.21	25.00	0.00	0.00	0.00
**41**	41.86	12.50	0.00	0.00	2.38
**43**	16.28	0.00	0.00	0.00	0.00
**44**	16.28	0.00	0.00	0.00	2.38
**46**	76.74	25.00	0.00	6.25	2.38
**47**	72.09	12.50	0.00	0.00	2.38
**51**	79.07	87.50	0.00	0.00	7.14
**55**	83.72	62.50	7.69	0.00	9.52
**62**	81.40	50.00	0.00	0.00	4.76
**65**	37.21	0.00	15.38	6.25	7.14
**72**	69.77	50.00	7.69	0.00	16.67
**75**	90.70	87.50	0.00	12.5	11.90
**82**	32.56	25.00	0.00	0.00	9.52
**89**	34.88	37.50	0.00	0.00	0.00
**96**	44.19	25.00	23.08	0.00	4.76
**99**	46.51	12.50	0.00	0.00	11.90
**109**	44.19	12.50	0.00	0.00	0.00
**125**	88.37	75.00	7.69	0.00	21.43
**131**	30.23	12.50	0.00	0.00	0.00
**143**	11.63	0.00	0.00	0.00	0.00
**152**	11.63	0.00	0.00	0.00	0.00
**163**	4.65	0.00	0.00	0.00	0.00

The polypeptide fractions with the highest frequency including 29, 31, 33, 47, 51, and 62 kDa fractions were detected using 81.4%, 65.1%, 72%, 72%, 79%, and 81.4% of the sera, respectively. These bands were not detected using the sera of the healthy participants from endemic and non–endemic areas for VL. Moreover, 19–23, 27, 31–35, 143–163, and 109 kDa fractions were detected specifically using the sera of the patients with VL (Table 1, Fig.2).

In the cutaneous leishmaniasis group, 100% of the samples recognized a minimum of 2 and maximum of 14 immunodominant bands. Bands with molecular weights 14–131 kDa were observed, of which more than 60% were 51 (87.5%), 55 (62.5%), 75 (87.5%), and 125 (75%) kDa (Table 1).

In the negative control group (G: III), i.e. serum samples of healthy individuals from endemic areas (n: 13) that were not reactive for VL in the DAT serologic test, 8 samples (61.54%) were negative for a diagnosis of VL by western blotting and no bands were observed. Bands with molecular weight 17, 55, 72 (similar frequency 7.69%), 65 (15.38%), 96 (23.08%) and 125 (7.69%) kDa were detected using 5 serum samples (38.46%) with a weak reaction (Table 1). One to three immunodominant bands were recognized in 38.46% of the samples.

In the other negative control group (G: IV) including serum samples of healthy individuals from non-endemic areas (n: 16) that were not reactive for VL in the DAT serologic test, 13 samples (81.25%) were negative for a diagnosis of VL by western blotting and no bands were observed. Following the reaction of 3 serum samples (18.75%), bands with molecular weights 17, 26, 46, 65 kDa (similar frequency 6.25%) and 75 kDa (frequency 12.5%) showed a poor reaction (Table 1). One band was recognized using 12.5% of the serum samples and 6.25% of the serum samples showed 4 bands.

In the other infectious or autoimmune diseases group (G: V), serum samples were negative for VL in the DAT serologic test. Twenty of these samples (47.62%) were also negative by western blotting, while in 22 samples (52.38%), the immunoreaction between 14–125 kDa bands and serum antibodies was weak (Table 1).

Chi-square showed that among all antigenic proteins, the frequency of 26 polypeptide fractions, i.e. 14–131 kDa fractions, had a significant difference (*P*<0.001) between VL and CL groups (G: I, II) versus other groups (G: III, IV, V). There was also a significant difference (*P*<0.001) in the frequency of 28 polypeptide fractions including 14–131 kDa fractions between the VL group (G: I) versus healthy controls and other diseases groups (G: III, IV, V). There was a significant difference (*P*<0.001) in the frequency of 28 polypeptide fractions including 17–131 kDa fractions between the VL group (G: I) versus other groups (G: II, III, IV, V).

A significant difference (*P*<0.05) was also observed in the band frequency between VL patients group (G: I) and CL patients group (G: II) for 7 polypeptide fractions including 29 (*P*=0.001), 31 (*P*=0.034), 33 (*P*=0.010), 35 (*P*=0.005), 46 (*P*=0.003), 47 (*P*=0.001), 65 (*P*=0.037) kDa fractions.

## Discussion

As a general rule, western blotting can be performed to diagnose different types of VL in humans and reservoir hosts (dogs) and also patients suffering from both HIV/AIDS and VL simultaneous, PKDL and post treatment follow-up and identifying carriers in endemic areas (18–20). In addition, this method can be used to evaluate the host-parasite interaction and recombinant antigen purity. Western blotting helps to assess the detection power of different antigens in specific strains and identify immunodominant antigens (18, 21, 22).

In this research, a profile of different immunodominant polypeptide fractions of Iranian *L. infantum* total antigens was observed using the sera of VL and CL patients, negative control subjects from endemic and non-endemic areas, and patients with other infectious diseases such as malaria and toxoplasmosis, etc. (Figs. 2, 3).

Among them, 85% of the proteins were detected as immunodominant bands using the sera of VL patients. This variety is because of the stimulation of B lymphocytes and antibody production in VL patients. Such various patterns are due to both the antigenic structure of the used parasite and also the patient’s genetic background which affects the immune response.

Thirty-four bands with molecular weights 14–163 kDa were observed in VL patients group. In a similar study using proteins from *Leishmania chagasi* / *Leishmania donovani*, 36 bands with molecular weights 14.5–123 kDa were observed in American visceral leishmaniasis patients group, and 23 bands with molecular weights of 18.5–115 kDa were observed in CL patients group (23).

The immunoblotting method was used on the *L. infantum* strain. Sera of VL and CL patients recognized numerous antigens with molecular weights ranging from 12–94 kDa and 14–68 kDa, respectively (24).

In western blotting, it is difficult to determine the molecular weight of the protein on the SDS-PAGE and various items like culture factors, the growth phase of the *Leishmania* parasite, the concentration and size of the SDS-PAGE, and the type of protein marker affect the determination of the molecular weight of polypeptide fractions. Moreover, the diverse results of most studies may be due to different strains of *Leishmania* and various protein preparation approaches (24–26).

In this study, most of the immunodominant polypeptide fractions had molecular weights 14–75 kDa (Table 1). The immunoblotting method was used on the *L. infantum* strain and serum samples of VL patients and reported that 14–16 kDa antigens had the highest specificity (27). Some immunodominant polypeptide fractions with high molecular weights (82–163 kDa) were observed. Such bands with molecular weights 119 and 123 kDa were observed in the reaction of VL patients’ sera with *L. infantum* antigens (23). Moreover, a similar study showed a 119kDa band (28).

In our study, 34 out of 40 *L. infantum* poly-peptide fractions were recognized as immunodominant proteins in reaction with VL patients’ sera. In the VL group, the most positive reaction was recognized in immunodominant polypeptide fractions with molecular weights of 75 (90.70%), 125 (88.37%), and 55 (83.72%) kDa.

Proteomic, mass spectrometry and immunoblotting techniques were used. The results showed immunodominant proteins with molecular weights 75 and 55 kDa in reaction between *L. infantum* promastigotes and VL patients’ sera (21). Considering their molecular weight and search for *Leishmania* parasite proteins in the Protein Data Bank (PDB), the proteins are probably related to heat-shock protein 70 kDa and histidine secretory acid phosphate, involved in parasite pathogenesis. Heat shock proteins (HSPs), also known as molecular chaperones, are highly conserved molecules that play important roles in protein folding, assembly of protein complexes, translocation of proteins across cellular compartments, and immunological developments (29, 30).

Moreover, in this study, proteins with molecular weights 31–53 kDa and 60–62 kDa were seen in more than 50% of the cases using the sera of VL patients. In a similar study, there was antibody against 63 kDa polypeptide fractions in the sera of 59 (96.7%) and against 32–35 kDa fractions in the sera of 51 (83.6%) CL patients (31).

These proteins are categorized as protein groups with effective roles in pathogenesis like elongation factors, chaperones; as well as tubulin and other housekeeping proteins (32). Chaperones are molecular components in the maintenance of *Leishmania* cellular homeostasis and survival, not only during stress but also under optimal growth conditions (33). From other proteins with more than 50% frequency, 16–17 kDa polypeptide fractions like ribosomal protein, P-21 protein, and hypothetical proteins, 27–30 kDa and 32–33 kDa proteins such as cysteine protease and calreticulin, 35 kDa proteins like phosphoglycan beta galactosyltransferase and surface antigen like protein, and 51 kDa proteins such as beta-tubulin can be mentioned. Moreover, the 70 kDa fraction is related to HSP 70 (21, 34).

Therefore, immunoblotting can help to identify immunogenic proteins to be used as biomarkers for diagnosis, treatment, and follow-up after confirmation with more advanced approaches like proteomics and mass spectrometry (35).

In this research, the maximum number of polypeptide fractions (28 bands) had a significant difference between VL and other groups, including negative control groups and other infectious disease, which may indicate the presence of specific antibodies in VL patients against these polypeptide fractions. These polypeptide fractions can be used as a biomarker for the diagnosis of VL.

We found a significant difference in the lowest number of polypeptide bands (6 bands) between visceral and cutaneous leishmaniasis groups. The reason for lack of any significant difference in immunodominant bands between VL and CL groups in most cases might be the high similarity of *L. infantum* antigenic proteins with *L. major* and *L. tropica* species.

The present study demonstrated that the immunoblotting technique does not have enough discriminatory power to differentiate VL from CL.

In addition, a significant difference was observed in some cases like 29–35, 46–47, 65 kDa polypeptide fractions that might be due to the presence of specific antigenic proteins in *L. infantum* or decreased humoral immune system stimulation resulting in the absence of antibody production against the above antigenic proteins in CL patients.

The recognized immunodominant polypeptide fractions in healthy subjects group from endemic and non-endemic areas may indicate the presence of cross-reaction between the antibody present in the patients’ sera and *L. infantum* polypeptides. The sera of the patients with viral and microbial diseases did not react with *L. infantum* antigens. Most of the cross-reactions were related to parasitic infections, especially toxoplasmosis and malaria detected in 72, 75, 99, and 125 kDa bands.

## Conclusion

As expected, some of the proteins identified in the present work were previously associated with humoral responses in VL and are candidate antigens for diagnosis. Western blotting would be the most ideal approach if it could distinguish positive sera from the sera of negative controls and patients with other infectious diseases. However; there is no special fraction to detect 100% of the disease cases correctly and more attempts are necessary. This technique could be a primary step for further exploration of VL immunodominant antigens detection by advanced techniques like proteomics approach for investigations in future planning.
